# Protective Effect of Probiotics on Cardiac Damage in Experimental Sepsis Model Induced by Lipopolysaccharide in Rats

**DOI:** 10.3390/medicina61040589

**Published:** 2025-03-25

**Authors:** Necip Gökhan Taş, Osman Aktaş, Hakan Gökalp Taş, Selim Zırh, Nezahat Kurt, Hakan Uslu

**Affiliations:** 1 Experimental Animal Application and Research Center, Erzincan Binali Yıldırım University, Erzincan 24002, Turkey; gokhantas7924@gmail.com; 2 Department of Medical Microbiology, Faculty of Medicine, Ataturk University, Erzurum 25030, Turkey; uhakan@atauni.edu.tr; 3 Department of Anesthesiology and Reanimation, Faculty of Medicine, Erzincan Binali Yıldırım University, Erzincan 24002, Turkey; hakangokalptas@hotmail.com; 4 Department of Histology and Embryology, Faculty of Medicine, Erzincan Binali Yıldırım University, Erzincan 24002, Turkey; szirh@erzincan.edu.tr; 5 Department of Medical Biochemistry, Faculty of Medicine, Erzincan Binali Yıldırım University, Erzincan 24002, Turkey; nezahat.kurt@erzincan.edu.tr

**Keywords:** cardiac injury, LPS, probiotic, sepsis, rat

## Abstract

*Background and Objective*: Probiotics have been shown to be effective in controlling various adverse health conditions such as antibiotic-associated diarrhea, inflammatory bowel disease, obesity, and neurological diseases. However, to our knowledge, there is no research on the preventive effect of probiotics on heart damage caused by infections. This study examined the preventive benefits of probiotics against sepsis-related heart injury using a rat model caused by lipopolysaccharide (LPS). *Materials and Methods*: Four groups of twenty-four male Wistar albino rats, each with six rats, were set up. For 14 days, Group 1 (Sham Group) was given oral normal saline, intraperitoneal *Escherichia coli* O111-B4 lipopolysaccharide (LPS Group) was given to Group 2, and oral probiotics were given to Group 3 (Probiotic Group). *Escherichia coli* O111-B4 lipopolysaccharide was injected intraperitoneally after Group 4 (Probiotic + LPS) received oral probiotics containing *Lactobacillus rhamnosus* GG and *Bifidobacterium animalis* subsp. *lactis* BB-12 (10^9^ CFU/day). Blood samples were taken twenty-four hours following the administration of LPS. The animals were then euthanized by cervical dislocation, and samples of cardiac tissue were taken in order to assess any damage to the heart. The following serum values were measured: C-reactive protein (CRP), creatine kinase-myocardial band (CK-MB), cardiac troponin subunit I (cTn-I), tumor necrosis factor-alpha (TNF-α), interleukin-1 beta (IL-1β), and interleukin-6 (IL-6). The TNF-α, IL-1β, IL-6, glutathione (GSH), malondialdehyde (MDA), Total Oxidant Status (TOS), Total Antioxidant Status (TAS), Oxidative Stress Index (OSI), CRP, CK-MB, and cTn-I levels were assessed in tissue samples. Additionally, staining techniques were used to analyze histopathological alterations in tissues. *Results*: With the exception of serum IL-6 (*p* = 0.111), tissue and serum cytokine levels were considerably greater in the sepsis group (Group 2) than in the other groups (*p* < 0.05 to <0.001). The TAS, GSH, and SOD levels were significantly lower (*p* < 0.05 to <0.001) in septic rats, although the tissue levels of TOS, OSI, and MDA were significantly higher. With the exception of serum CRP in Group 3 (*p* = 0.328), the CK-MB, CRP, and cTn-I levels were considerably higher in Group 2 than in the other groups (*p* < 0.01 to <0.001). When compared to the other groups, histopathological examination showed significant alterations in the LPS group. *Conclusions*: Probiotics showed positive effects on oxidative stress markers and dramatically decreased sepsis-induced cardiac damage in the LPS-induced sepsis model. These results imply that probiotics could be used as a therapeutic approach to lessen the cardiac damage brought on by sepsis.

## 1. Introduction

As a complicated and sometimes fatal disease, sepsis—also referred to as septicemia or blood poisoning—can result in abrupt organ failure and tissue damage, frequently leading to high death rates among those who have the disease [1,2,3]. Because of its high rates of morbidity and mortality worldwide, sepsis is a major global health concern and one of the World Health Organization’s top priorities [4,5,6]. For example, sepsis is estimated to be the leading cause of in-hospital fatalities in the United States, with an annual cost impact of over USD 24 billion [5]. With in-hospital death rates of 25–30%, sepsis-related hospitalizations outnumber those caused by myocardial infarction and stroke [7]. Patients frequently experience long-term health problems including decreased quality of life, cognitive impairment, chronic kidney disease, and an elevated risk of long-term mortality, even if they survive sepsis [8].

The equilibrium between pro- and anti-inflammatory responses becomes disrupted in sepsis. The release of anti-inflammatory cytokines, aberrant immune effector cell death, and the hyperproliferation of immunosuppressive cells are important indicators of sepsis [9]. Cytokine expression, cytokine storms, and excessive reactive oxygen species (ROS) and reactive nitrogen species (RNS) synthesis all contribute to the inflammatory process. Enzymes like myeloperoxidase and NADPH oxidase are activated during inflammation, and the process of producing ROS also aids in eliminating bacteria. High mortality rates, however, can result from excessive ROS/RNS generation, which can impair mitochondrial function and cause cell death by necrosis, apoptosis, and pyroptosis [3].

Uncontrolled inflammation during sepsis causes ROS/RNS to destroy tissue, which may result in immune system malfunction and multiple organ failure. The body’s antioxidant defense systems destroy ROS/RNS, reducing inflammation and encouraging tissue regeneration, whereas oxidants worsen inflammation and tissue damage [10]. Free radicals produced during sepsis have the ability to harm proteins, membrane lipids, and DNA, which can lead to cellular malfunction and post-sepsis syndrome. Long-term issues like cognitive and psychological impairments, protracted medical treatments, and a higher risk of readmission are characteristics of this syndrome [11,12].

Since oxidative stress is a major factor in septic shock, ischemia–reperfusion injury, multiple organ failure, and acute respiratory distress syndrome, antioxidants have been proposed as a possible treatment for this condition [13]. A promising solution to these issues is to use probiotics, which are non-pathogenic microorganisms that are naturally found in healthy people’s gastrointestinal tract and mucosa. These microorganisms are extremely resilient to a variety of challenging circumstances, such as those that are oxygenated, bile-rich, and acidic [14]. By assisting with vitamin production, protein digestion, and mineral absorption, probiotics promote the health of the host. Additionally, they alleviate disorders including hepatic steatosis, insulin sensitivity, lactose intolerance, and blood cholesterol levels [15]. The most commonly used probiotic bacteria in children and adults for treatment purposes are *Lactobacillus rhamnosus* GG and *Bifidobacterium animalis* subsp. *lactis* BB-12 [16,17,18,19,20]. We also preferred these organisms in our study.

Probiotics have anti-inflammatory, immuno-modulatory, anticarcinogenic, and antihypertensive properties in addition to their metabolic advantages [21,22,23]. Antibiotic-associated diarrhea, inflammatory bowel disease, obesity, and even neurological diseases can result from the unchecked use of antibiotics, especially those given for infections, which disrupts the balance of gut microbiota. Probiotics are thought to be useful in reducing and reversing these adverse circumstances [24].

In this context, our objective was to examine the potential of probiotics to prevent sepsis-induced cardiac damage in vivo, utilizing both biochemical and histopathological analyses. By investigating an area previously unexplored, we aimed to provide a meaningful contribution to the scientific literature.

## 2. Materials and Methods

This study, which investigates the functionality and therapeutic efficacy of probiotics containing *Lactobacillus rhamnosus* GG and *Bifidobacterium animalis* subsp. *lactis* BB-12 on cardiac damage, was conducted in collaboration between the Department of Medical Microbiology, Faculty of Medicine, Atatürk University, and the Experimental Animal Research and Application Center, Erzincan Binali Yıldırım University. This study was conducted in accordance with the 3R (Replacement, Reduction, and Refinement) regulation regulating the ethical rules of animal experiments. Therefore, the number of animals to be used was kept as small as possible without compromising the project objectives. The “tradition or common sense” approach method was used when determining the sample size.

### 2.1. Grouping the Rats and Experimental Applications

Male albino Wistar rats, aged 8–12 weeks and weighing between 200 and 250 g, were acquired and allowed to acclimatize for one week to facilitate their adaptation to the laboratory environment. The animals were maintained at a temperature of 22 ± 2 °C, with a 12 h light/dark cycle and 50–60% humidity. They were provided with standard laboratory chow and water ad libitum. During this process, contact and drug application exercises were performed to prevent stress symptoms in the future oral gavage application.

The rats were randomly divided into four groups, with six rats in each group:

1. Sham Group (SG): this group was administered 0.5 mL of normal saline (0.9% NaCl) daily via oral gavage for 14 days;
2. Probiotic Group (PG): this group received 0.5 mL of probiotics (25 mg/kg, 10^9^ CFU/day) daily via oral gavage for 14 days;
3. LPS Group (LPSG): this group served as the sepsis model, receiving lipopolysaccharide (LPS) administration;
4. Probiotic + LPS Group (PLPSG): this group was treated with probiotics as described for PG and received LPS on day 14.

In our experiment, each sachet of Forbiome Probiotic (FP) (Forbiome^®^ from Chr Hansen, Abdi Ibrahim, Istanbul, Turkey) contained 10 billion probiotic microorganisms, including 300 mg of inulin, 47 mg of *Lactobacillus rhamnosus* GG, and 20 mg of *Bifidobacterium animalis* subsp. *lactis* BB-12^®^. At the end of day 14, a single intraperitoneal dose of 5 mg/kg of *E. coli* O111 was administered to the LPSG and PLPSG.

### 2.2. Anesthesia Procedure

At the conclusion of day 14, the rats were anesthetized via intraperitoneal injection of a mixture containing 40 mg/kg of ketamine hydrochloride (Ketalar, Pfizer, Inc., New York, NY, USA) and 10 mg/kg of xylazine (Rompun; Bayer Health Care AG, Leverkusen, Germany). Blood samples were drawn from the jugular vein of anesthetized rats into both anticoagulated and non-anticoagulated tubes. Following anesthesia, cervical dislocation was performed, and the animals were euthanized while positioned on their backs, after which heart tissues were harvested.

### 2.3. Acquisition, Preservation, and Preparation of Serum and Tissue Specimens for Analysis

Blood samples were centrifuged at 1500× *g* for 10 min at +4 °C, and the serum phase was carefully collected into Eppendorf tubes. After euthanasia, half of the heart tissue samples were placed in clean Petri dishes for biochemical analysis, while the other half were fixed in 10% neutral formaldehyde solution for histopathological examination. Serum and heart tissue samples were stored at −80 °C until further analysis. All tissue and serum samples were allowed to reach room temperature on the day of analysis. From the tissue samples, 50–100 mg was extracted, and 2 mL of ice-cold phosphate-buffered saline (50 mM, pH 7.4) was added before homogenization on ice. The homogenates were then centrifuged at 10,000× *g* for 15 min at 4 °C, and the supernatants were collected.

### 2.4. TNF-α, IL1β, and IL6 Analysis in Serum and Tissue Samples

Enzyme-linked immunosorbent assays (ELISAs) were utilized to determine the levels of TNF-α, IL-1β, and IL-6 in the serum and heart tissue of rats. To ensure accuracy, each test was repeated three times, and the average of the three measurements was considered. In this study, ELISA kits from SunRed Biotechnology Company (Shanghai, China) were employed to measure TNF-α, IL-1β, and IL-6 levels (Rat TNF-α, Lot: 202403; Rat IL-6 and Rat IL-1β, Lot: 202401014). All the test procedures and result interpretations strictly followed the manufacturer’s instructions.

### 2.5. CRP, CK-MB, and cTn-I Analysis in Serum Samples

ELISA was used to measure the levels of C-reactive protein (CRP) and creatine kinase muscle and heart isoenzyme (CK-MB) in serum samples. The Bioassay Technology Laboratory provided the BT-LAB kits for CRP (Rat C-Reactive Protein, CRP/PTX1) and CK-MB (Rat CK-MB). Following the instructions in the kits, both tests were performed using the reagents provided. For cTn-I measurement, a chemiluminescent immunoassay (CMIA) was conducted using the Abbott Architect i2000 device (Abbott Laboratories, Abbott Park, IL, USA). All the test procedures and result interpretations adhered to the manufacturer’s guidelines.

### 2.6. MDA, GSH, SOD, TOS, and TAS Analysis in Tissue Sample

Malondialdehyde (MDA) was measured using the Thiobarbituric Acid Reactive Substance (TBARS) Test Kit (Lipid Peroxidation Test Kit, Antibodies, Lot: 43016). This kit is a quantitative test for the colorimetric measurement of MDA in various samples such as animal and plant tissues, serum, and plasma. The principle of the test is based on the colorimetric measurement of the red color produced by the reaction of MDA, a breakdown product of peroxidized lipids in serum, with thiobarbituric acid (TBA) under acidic and high-temperature conditions (90–100 °C).

Reduced glutathione (GSH) was measured using the Glutathione Colorimetric Test Kit (Elabscience^®^ Biotechnology Inc., Chengdu, China, Cat. No: E-BC-K030-M, Cayman). Initially, all the samples were treated with 1:1 meta-phosphoric acid to deproteinize them. After the centrifugation of the supernatant, the samples were used for analysis.

Superoxide dismutase (SOD) is one of the primary defense mechanisms against free radicals in cells and plays a role in preventing cellular damage. The total SOD analysis was performed using the Total Superoxide Dismutase (T-SOD) Activity Test Kit (Hydroxylamine Method, Cat. No.: E-BC-K019-M, Elabscience^®^, Cayman), which measures the three isoforms of SOD (Cu/Zn, Mn, and FeSOD).

The TOS (Total Oxidant Status) measurement was carried out using the TOS Colorimetric Test Kit (Elabscience^®^ Biotechnology Inc., Cat. No: E-BC-K802-M, Rel Test). In this test, oxidants in the sample oxidize the iron ion chelator complex into iron ions. The ferric ions form a colored complex with a chromogen in an acidic environment. The results were evaluated by spectrophotometric analysis of the color change at 660 nm.

The TAS (Total Antioxidant Status) measurement was carried out using the TAS Colorimetric Analysis Kit (E-BC-K801-M, Elabscience Biotechnology Co., Ltd., Wuhan, China). In this test, antioxidants in the sample convert the dark blue-green ABTS radical to the colorless ABTS form. The amount of TAS in the sample was determined by measuring the absorbance of the colored ABTS at 660 nm.

All of the aforementioned tests were conducted and interpreted in accordance with the manufacturer’s instructions for each kit.

### 2.7. Calculation of Oxidative Stress Index

The Oxidative Stress Index (OSI) value was calculated using the formula TOS (μmol H_2_O_2_ equivalent/L)/TAS (mmol Trolox equivalent/L), as described in the literature. The OSI results are expressed as percentages.

### 2.8. Histopathological Analysis

Immediately after the animals were sacrificed, cardiac muscle tissues were placed in a 10% buffered formaldehyde solution and incubated at room temperature for two days. Following fixation, the tissues were processed using an automated tissue processor (Leica TP1020, Leica Biosystems Nussloch GmbH, Nussloch, Germany) and embedded in paraffin. Subsequently, the tissues were blocked using a paraffin embedding station (Leica EG1150 H, Leica Biosystems Nussloch GmbH, Nussloch, Germany). From the paraffin-embedded blocks, four-micrometer-thick sections were cut using a rotary microtome (Leica SM2010 R, Leica Biosystems Nussloch GmbH, Nussloch, Germany) and mounted onto slides. After deparaffinization, the sections were rehydrated through a graded alcohol series. Five sections were obtained from each tissue block for each staining method (hematoxylin–eosin and Masson’s trichrome staining), resulting in a total of ten sections per block. Trichrome staining was performed following the instructions provided by the commercial staining kit.

To enhance staining quality, the slides were re-fixed in Bouin’s solution at 56 °C for one hour after preservation. The slides were rinsed under running water for five minutes to remove the yellowish coloration. They were then stained with Weigert’s iron hematoxylin solution for ten minutes, and any excess stain was removed by washing the slides under running warm tap water for ten minutes. Subsequently, the sections were stained with Biebrich scarlet-acid fuchsin solution for ten minutes and washed in distilled water to remove residual dye.

The sections were then immersed in a phosphomolybdic and phosphotungstic acid solution for ten minutes or until collagen fibers were no longer stained red under light exposure. Without rinsing, the sections were immediately submerged in an aniline blue solution for five minutes to stain the collagen fibers. After staining, the slides were rinsed with distilled water and placed in a 1% acetic acid solution for two minutes. Finally, the slides were washed again in distilled water to complete the process. Finally, following the standard mounting protocol, slides were sequentially immersed in 95% ethanol, 99% ethanol, and xylene before being sealed with coverslips using a resin-based mounting medium. Once fully dried, the slides were set aside until microscopic examination. To achieve nuclear staining, the preparations were stained with Harris hematoxylin for five minutes, followed by rinsing under running water for five minutes. For cytoplasmic staining, the slides were immersed in an Eosin Y solution for two minutes before counterstaining. The preparations were then gently dipped in 96% ethanol five times, followed by immersion in 100% ethanol for two minutes. Finally, three sequential xylene treatments, each lasting two minutes, were applied to remove excess stain from the tissue. Coverslips were mounted using Entellan medium to protect the tissue surfaces.

For each preparation, five random regions were selected and imaged using an Olympus BX43 Upright Microscope and an Olympus Digital Microscope Camera (DP22) (Olympus Corporation, Tokyo, Japan). The captured images were analyzed for counting, measurement, and scoring using ImageJ software (version 18.0, NIH, Bethesda, MD, USA).

### 2.9. Statistical Analysis

Statistical analyses were conducted using the Statistical Package for Social Sciences (SPSS) software version 22.0 for Windows (IBM Corp., Armonk, NY, USA). Results for continuous variables are expressed as the mean ± standard deviation (SD). One-way analysis of variance (ANOVA), a parametric test, was used to determine significant differences between multiple groups. Following this test, a post hoc test was carried out to determine which groups were different from each other. A *p*-value of less than 0.05 was considered statistically significant.

## 3. Results

### 3.1. Biochemical Findings in Tissue and Serum

**Figure 1:**
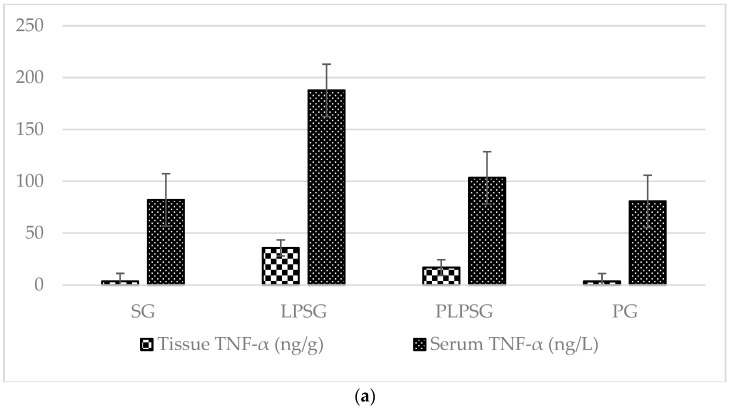

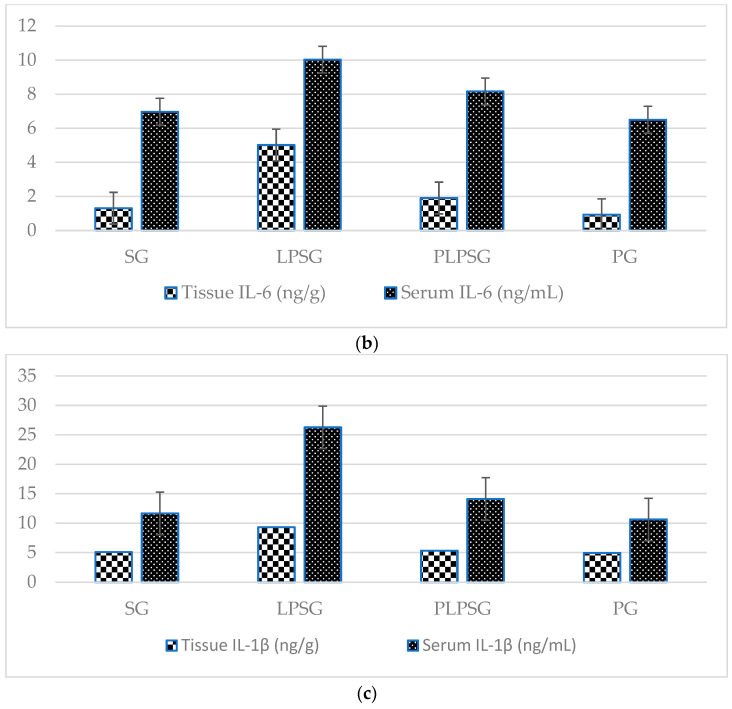
A comparison of cytokine concentrations in tissue and serum samples across rat groups. (**a**) Tissue and serum concentrations of TNF-α in the rat cohorts. (**b**) Concentrations of IL-6. (**c**) The levels of IL-1β in the respective groups.

The cytokine levels (TNF-α, IL-6, and IL-1β) in tissue and serum samples obtained from different rat groups are shown in Figure 1 and Table 1. The levels of TNF-α, IL-6, and IL-1β in both tissue and serum were significantly higher in the LPSG compared to the other groups (*p* < 0.05 to <0.001), except for the serum IL-6 level, which was similar between the LPSG and PLPSG. The cytokine levels in both tissue and serum were compared between the SG and PG. In the PLPSG, the cytokine levels were similar to those in the SG and PG, except for the tissue TNF-α level, which was significantly higher in the PLPSG compared to that in the SG and PG.

**Figure 2:**
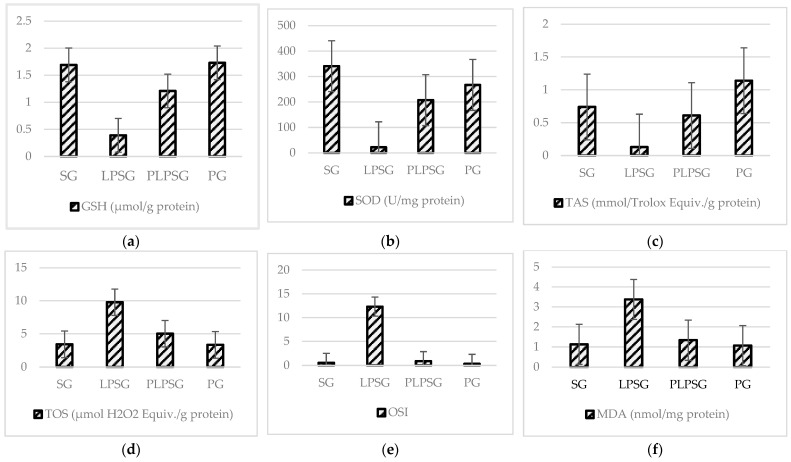
While (**a**) GSH, (**b**) SOD, and (**c**) TAS levels were found to be significantly lower in the LPSG compared to the other groups, (**d**) TOS, (**e**) OSI, and (**f**) MDA levels were found to be significantly higher in the LPSG.

Figure 2 and Table 2 present a comparison of the TAS, TOS, OSI, MDA, GSH, and SOD protein levels from tissue samples based on the rat groups. In the LPSG, the TAS, GSH, and SOD levels were lower than in the other groups, while the TOS, OSI, and MDA levels were higher. The PLPSG and PG demonstrated increasing or decreasing values identical to the SG in all biochemical assays. In the SG, the TAS, GSH, and SOD values were significantly higher compared to those in the PLPSG (*p* < 0.01 to <0.001), while the TOS, OSI, and MDA values were significantly lower (*p* < 0.05 to <0.001). No significant difference was observed between the SG and PLPSG values in any of the biochemical tests. The lower TAS value in the SG compared to the PG was found to be significant (*p* < 0.05). In the LPSG, the TAS, OSI, and MDA values were higher compared to those in the PLPSG and PG (*p* < 0.05 to <0.001), while the TAS, GSH, and SOD values were lower (*p* < 0.05 to <0.001). In the PLPSG, the TAS and GSH values were significantly lower compared to those in the PG.

### 3.2. Findings in Serum CK-MB, CRP, and Troponin I

**Figure 3:**
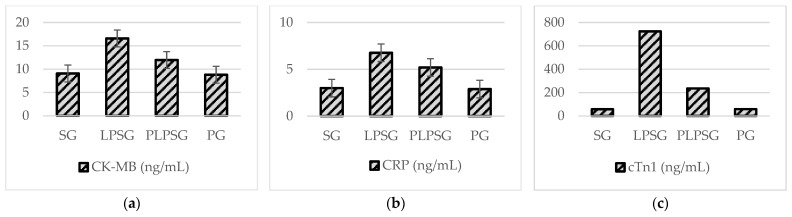
A comparison of biochemical test results applied to tissues based on rat groups: (**a**) the CK-MB level was found to be the highest in the LPSG, followed by the PLPSG, and was lower in the SG and PG. (**b**) Similar characteristics were observed in the CRP level. (**c**) Although a similar situation was observed in the troponin 1 level, troponin levels in the PLPSG, SG, and PG showed a sharp decrease compared to those in the LPSG.

Figure 3 and Table 3 present a comparison of serum CK-MB, CRP, and cTn-I levels among rat groups.

Table 3 shows the statistical significance of the findings from comparing CK-MB, CRP, and cTn-I in the rat groups: CK-MB levels were found to be significantly higher in the LPSG compared to those in the SG (*p* < 0.001), PLPSG (*p* < 0.01), and PG (*p* < 0.001). CK-MB levels in the PLPSG were not substantially different from those in the SG (*p* = 0.147) or the PG (*p* = 0.098). We found similar CK-MB levels between the PG and the SG (*p* = 0.996). We found that the LPSG’s CRP level was significantly higher than that of the SG and PG (*p* < 0.01). The difference between the LPSG and the PLPSG was not significant (*p* = 0.328). CRP levels in the PLPSG were not substantially different from those in the SG (*p* = 0.096) or the PG (*p* = 0.077). The levels of the PG and the SG were similar (*p* = 0.999). When the serum troponin I levels were examined, it was found that those in the LPSG were significantly higher than those in the SG, PLPSG, and PG (*p* < 0.001). The level in the PLPSG was similar to those in the SG and PG (*p* = 0.179, *p* = 0.184, respectively). We found that the PG’s troponin I level was similar to that in the SG (*p* = 1.000).

### 3.3. Histopathological Findings

**Figure 4:**
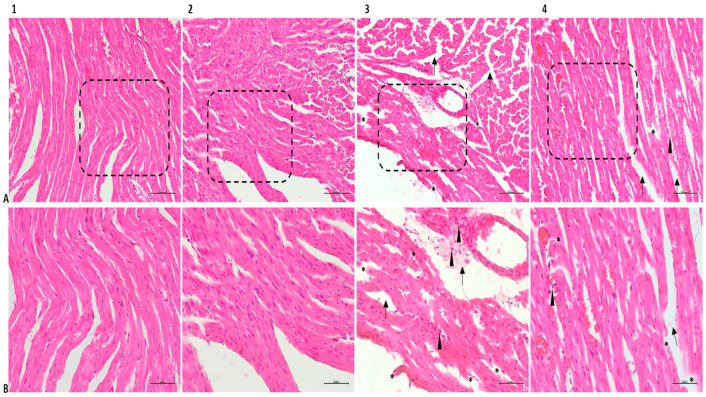
H&E-stained micrographs of heart tissues in experimental groups. Numbers 1, 2, 3, and 4 correspond to SG, PG, LPSG, and PLPSG. (**A**) ×200 magnification. (**B**) ×400 magnification. Asterisks indicate fiber rupture, arrows indicate edematous areas, and arrowheads indicate infiltrative cell accumulations.

Hematoxylin and eosin (H&E) staining of the heart tissue samples of the SG and PG revealed healthy collaterals reflecting the natural histological appearance and oval nuclei located in the middle of the cytoplasm. In Masson trichrome samples, there were limited collagen-rich areas near the outer walls of the tissue. The LPSG showed significant pathological differences compared to the other groups. In tissue sections, widespread fibrillar breaks, increased cardiomyocyte diameters, and mononuclear cell infiltration were observed due to excessive metabolic activity resulting from infection (Figure 4).

**Figure 5:**
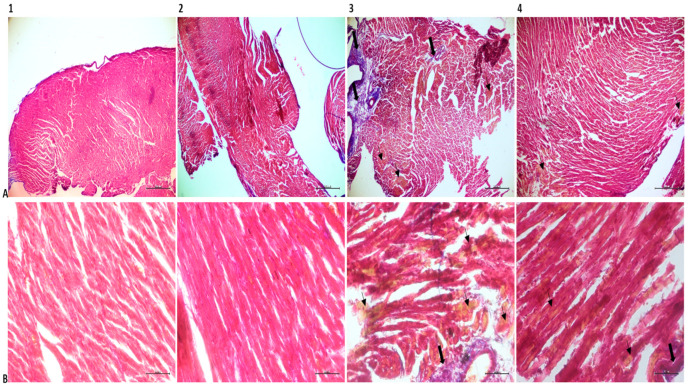
Masson’s trichrome-stained micrographs of heart tissues. Numbers 1, 2, 3, and 4 correspond to SG, PG, LPSG, and PLPSG. Line (**A**), ×40 magnification; line (**B**), ×400 magnification. Thick arrows indicate areas of connective tissue expansion; thin arrows indicate congestive deposits.

Tissue sections of the LPSG stained with Masson’s trichrome show a marked increase in the distance between muscle fibers, reflecting edema, high rates of collagen deposition, and marked accumulations of yellow-stained erythrocytes. There appears to be a decrease in histopathological changes in the PLPSG compared to the LPSG. The PLPSG had higher scores than the SG did but differed from the LPSG in that there were fewer signs of edema, the fibrillar structure remained the same, and there were few areas of infiltration on H&E sections. Although erythrocyte accumulations were prevalent in Masson’s trichrome sections in the PLPSG, they were significantly less than in the LPSG and had reduced collagen deposits (Figure 5).

**Figure 6:**
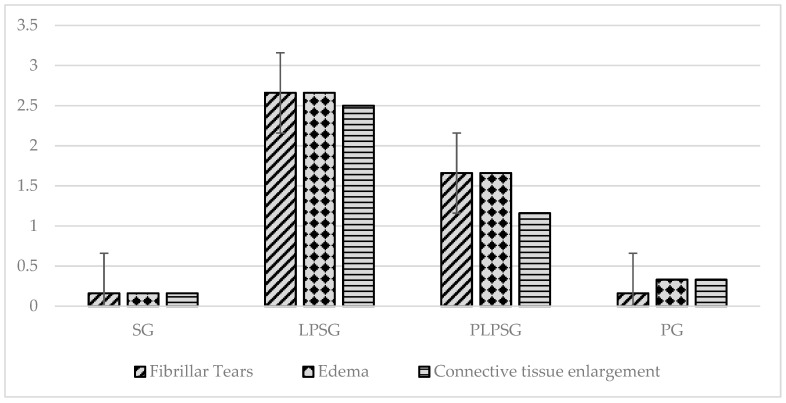
A comparison of histopathological scoring results in the heart tissues of the rat study cohorts. Histopathological examination revealed the most significant fibrillar tears in the LPSG, followed by the PLPSG, while the SG and PG exhibited the same minimal rate of fibrillar tears. Edema was highest in the LPSG and PLPSG, third in the PG, and last in the SG. The edema in the rat groups was observed to follow a similar order to the level of increase and expansion in the connective tissue.

Figure 6 compares the histopathological scoring results in heart tissues of rat groups, and Table 4 summarizes the statistical analysis of these results. In tissue samples, the LPSG and PLPSG had considerably larger fibrillar tears, edema, and connective tissue expansion than the SG and PG did (*p* < 0.001). However, the rate of histopathological findings was also significantly higher in the LPSG than in the PLPSG (*p* < 0.01 in fibrillar tears, *p* < 0.05 in edema, and *p* < 0.001 in connective tissue expansion). However, no significant difference was observed between the SG and PG in terms of fibrillar tears, edema, and connective tissue expansion (*p* = 1.000, 0.935, and 0.928, respectively).

## 4. Discussion

In sepsis, pathogens spread from the primary site of infection to numerous important organs, including the heart, brain, and lungs, causing considerable damage. Common symptoms of sepsis include tachycardia, hypotension, loss of consciousness, jaundice, metabolic acidosis, and anuria [25,26,27]. Healthcare workers have a critical responsibility to prevent the catastrophic effects of heart disease, which affects a vast population worldwide and has a high mortality rate. Clinical and laboratory research will aid in the fight against these diseases. In this laboratory-based investigation, we found that probiotics had a favorable influence on oxidative stress parameters in a lipopolysaccharide-induced rat sepsis model, resulting in a significant reduction in sepsis-induced heart muscle damage.

Our investigation indicated that the LPSG had significantly higher levels of IL-1β, IL-6, and TNF-α in tissue and serum than the SG, PLPSG, and PG did. The histopathological evaluation of cardiac tissues revealed significant pathological differences between the LPSG, SG, PLPSG, and PG. In addition to widespread fibrillary ruptures in H&E sections, cardiomyocyte diameters increased due to elevated infection-induced metabolic activity in the LPSG. Again, this group had the highest rate of mononuclear cell infiltration. Furthermore, the increase in the spacing between muscle fibrils reflecting edema, as well as the rapid rate of collagen deposition in LPS-treated Masson trichrome sections, is notable.

When the body’s oxidant–antioxidant balance shifts in favor of oxidants, it causes a wide range of pathological changes, including cell and tissue destruction. The homeostatic mechanisms that govern the pro- and antioxidant balance alter it [28]. Lipid peroxidation is one of the signs of high oxidative stress. ROS-induced lipid peroxidation is required for cell death, such as apoptosis, autophagy, and ferroptosis. This fundamental and conserved process is based on excessive ROS, which assault biomembranes, propagate lipid peroxidation chain events, and ultimately induce various types of cell death [29]. MDA is a molecule produced by the peroxidation of polyunsaturated fatty acids that is a byproduct of lipid peroxidation and an indicator of oxidative stress [30]. MDA and other oxidation products are formed when the membrane suffers oxidative damage. MDA levels and apoptosis counts indicate the transition of potentially malignant diseases to malignancy and can thus be used to predict oral epithelial dysplasia [31]. One study reported that LPS administration led to a significant increase in MDA levels in the mouse brain and liver compared to a control group that received only phosphate-buffered saline injections [32]. Another study comparing the efficiency of trimetazidine treatment to the experimental sepsis model discovered that MDA levels were considerably greater in the LPSG than in the control group [33]. In our study, MDA levels in the tissue were considerably greater in the LPSG compared to the SG, PG, and PLPSG, as in prior investigations.

CTn-I, CK-MB enzyme, and MDA levels in tissue and blood increase during heart muscle injury; these indications are essential for detecting suspected acute myocardial infarction [34]. In a study investigating the potential role of melatonin in ameliorating myocardial ischemia–reperfusion injury in transplanted hearts in a rat model, it was reported that melatonin significantly balanced the increase in TNF-α and IL-1 in myocardial levels and significantly reduced the severity of cardiac injury in rats undergoing a transplantation procedure [35]. In a study in which rats were subjected to prolonged exercise, the CTn-I enzyme level increased considerably, indicating a high risk of acute myocardial infarction [36]. In another study, it was reported that cisplatin increased MDA, CK-MB, and cTnI levels and inflammatory cytokines (TNF-α, IL-6, and IL-1β) in cardiac tissue, while decreasing GSH and catalase levels; on the other hand, oleuropein improved cardiac parameters and reduced inflammatory cytokine and oxidative stress levels in cardiac tissue [37]. In our study, the LPSG had considerably greater CK-MB and CTn-I enzyme levels than the SG, PG, and PLPSG did.

CRP, an acute phase protein produced by gene transcription in response to proinflammatory cytokines IL-6, TNF-alpha, and IL-1β in the liver, plays a crucial role in bacterial elimination. Acute infections with tissue damage, rheumatological diseases, autoimmune diseases, neurodegenerative disorders, malignancies, and similar tissue damage can cause a 10–100-fold increase in CRP levels (<1 mg/dL) within 6–72 h [38]. In our investigation, the CRP enzyme level was considerably greater in the LPSG than in the SG and PG, but not significantly higher than in the PLPSG.

Syringic acid, an antioxidant, is said to increase GSH content and the activities of SOD, catalase, glutathione peroxidase (GPx), glutathione-S-transferase (GST), and glutathione reductase (GR) in the isoproterenol-induced myocardial infarction rat model, as well as show cardioprotective effects by scavenging reactive oxygen species [39]. In our investigation, GSH levels in tissues were shown to be lower in the LPSG than in the SG, PG, and PLPSG. SOD levels in tissues were lower in the LPSG and PLPSG than in the SG and PG. TOS arises as a result of excess free radicals or insufficient antioxidants, and it destroys biological components such as DNA, lipids, and proteins [40]. A study investigating the levels of TAS, scube-1, and ischemia-modified albumin in experimentally produced acute pancreatitis in rats found no significant difference in TAS and TOS levels between the cerulein-induced and control groups [41]. In our investigation, the TAS level in the tissue was shown to be lower in the LPSG than in the PLPSG but higher in the SG and PG. The TOS level in the tissue did not differ significantly between the PG and the LPSG, but it was higher than in the SG and PLPSG. The LPSG had a similar OSI score to that of the PG, but it was greater than those of the SG and PLPSG.

Probiotics are organisms that help their hosts by lowering the amount of pathogenic bacteria, altering microbial metabolism, and boosting the immune system. Probiotics take three steps: first, in the intestinal lumen; then, in the intestinal mucus layer, epithelial cells, and subepithelial area; and finally, outside the intestines. The intestines contain microorganisms that have the ability to actively engage with the host immune system, boosting antitumor immune responses and lowering immunotherapy toxicity [42]. Lactobacillus and Bifidobacterium bacteria are the most common helpful microorganisms found in the colon. For a long time, many studies have been conducted on the beneficial effects of *L. rhamnosus* on the human body, including the effect on heart damage that we explored in our study. A 2008 study found that *L. rhamnosus* supplementation may be useful in avoiding the development of eczema in breastfed babies at risk of allergic illness [43]. An early study found that obese children with non-alcoholic fatty liver disease who received probiotic medication had significantly lower levels of alanine aminotransferase and anti-peptidoglycan-polysaccharide antibodies than those who received a placebo [44]. Many studies have revealed that probiotics regulate the release of cytokines and activate the secretion of IgG antibodies [45,46]. It is known that probiotics prevent the colonization of tissues by pathogens by producing antimicrobial substances and competing for adhesion receptors, by increasing mucosal secretion and the production of anti-inflammatory cytokines in the intestine, and by synthesizing neurotransmitters such as serotonin, dopamine, and gamma aminobutyric acid [47].

The preventive impact of probiotics, specifically *L. rhamnosus*, on heart injury in sepsis models has not been studied in vivo. Probiotics containing *L. rhamnosus* were administered to the PLPSG and PG for 14 days as part of our investigation. TAS levels in the tissue were significantly greater in the PG than in the SG. Probiotics raise the antioxidant level in the tissue, as seen by the rise in TAS. There was no histopathological difference between the tissues of the PG and SG. TNF-α, IL-6, and IL-1β levels in the tissue were found to be significantly lower in the PLPSG than in the LPSG. These findings demonstrated that probiotics lower the amount of proinflammatory cytokines in septic cardiac tissue. The tissue had mild but noticeable amounts of TOS and MDA. These findings demonstrate that probiotics lower MDA and total oxidant levels in cardiac tissue. The tissue has a low and noteworthy OSI value. According to these findings, probiotics increase antioxidants and decrease oxidants in heart tissue, lowering the ratio of total oxidants to antioxidants. The tissue has high and noteworthy amounts of TAS, GSH, and SOD. These findings demonstrate that probiotics raise the levels of total antioxidants in cardiac tissue in addition to antioxidants like SOD and GSH. Serum levels of TNF-α and IL-1β are low and considerable, while those of IL-6 are both low and significant. These findings demonstrate that probiotics lower serum levels of the proinflammatory cytokines TNF-α and IL-1β. Serum levels of CTn-I and CK-MB are both low and significant. By identifying low levels of CK-MB and CTn-I enzymes, which are serum markers of heart damage and directly relate to the goal of our study, these data offer encouraging evidence that probiotics help lessen heart damage in sepsis. Histopathological examination of cardiac tissue samples showed that the SG and PG had the same minimum pathologies, while the most significant pathologies occurred in the LPSG. Biochemical analyses showed a significant increase in CK-MB, CRP, and cTn1 levels in the LPSG compared to the SG and PG. Increased blood levels of these three markers are an important criterion for the diagnosis of myocardial infarction in humans. Similarly, increased levels of these markers in the blood of rats can be evaluated as an indicator of cardiac damage. In contrast to the LPSG, the PLPSG exhibits minimal and noteworthy histopathological alterations, such as fibrillar rips, edema, and connective tissue increases.

Our study has shown that *Lactobacillus rhamnosus* GG and *Bifidobacterium animalis* subsp *lactis* BB-12 may be useful in the treatment of heart damage. Currently, the efficacy and application methods of probiotics are being investigated in a wide range of diseases. We can say that the potential use of probiotics with various combinations of different probiotic bacteria will continue to be a subject of research for many years to come.

## 5. Conclusions

Treatments for sepsis can help lessen the disease’s symptoms to some extent, but they may also cause irreversible harm to organs and tissues. Probiotics and sepsis have been the subject of numerous studies in recent years. There are not many in vivo studies, though, looking into how probiotics can protect injured organs and tissues in sepsis model trials. This study demonstrates how probiotics can prevent cardiac muscle damage in sepsis. Our study’s findings indicate that probiotics hold promise as a substitute strategy for preventing cardiac disease. Probiotics have fewer adverse effects and lower production costs than other chemicals because they are part of the natural flora. Given the expenses associated with illnesses brought on by damage to the heart muscle, it is believed that a healthy heart will improve worker productivity and personal well-being while also making significant financial contributions through the protective effects of probiotics. Our demonstration that probiotics have a healing role in sepsis-induced heart damage in a preclinical study has offered an alternative solution to sepsis treatment in clinical units. Since probiotics are taken orally like nutrients and do not cause any harmful effects on the organism, we predict that clinicians will prefer probiotic administration in cases of suspected heart damage. Thus, it will be possible to evaluate the efficacy of probiotics in human sepsis patients.

## Figures and Tables

**Table 1:** Comparison of tissue TNF-α, IL-6, and IL-1β levels and comparison between groups.

Biomarkers	Rat Groups				Pairwise Comparison *p*-Value					
	SG (1)	LPSG (2)	PLPSG (3)	PG (4)	1 vs. 2	1 vs. 3	1 vs. 4	2 vs. 3	2 vs. 4	3 vs. 4
IN TISSUE										
TNF-α (ng/g)	3.60 ± 1.18	35.75 ± 8.21	16.85 ± 9.04	3.49 ± 2.02	<0.001	<0.01	1.000	<0.001	<0.001	<0.01
IL-6 (ng/g)	1.31 ± 1.39	5.02 ± 2.90	1.91 ± 0.30	0.93 ± 0.78	<0.05	0.922	0.978	<0.05	<0.05	<0.01
IL-1β (ng/mg)	5.08 ± 0.43	9.31 ± 4.83	5.32 ± 0.25	4.94 ± 0.29	<0.05	0.998	1.000	<0.05	<0.05	0.993
IN SERUM										
TNF-α (ng/L)	82.06 ± 6.80	187.68 ± 86.55	103.40 ± 16.24	80.69 ± 15.29	<0.01	0.842	1.000	<0.05	<0.01	0.816
IL-6 (ng/L)	6.97 ± 1.46	10.03 ± 1.61	8.16 ± 1.10	6.50 ± 1.17	<0.01	0.445	0.931	0.111	<0.01	0.181
IL-1β (ng/mL)	11.66 ± 0.77	26.27 ± 6.68	14.11 ± 3.02	10.62 ± 1.32	<0.001	0.675	0.963	<0.001	<0.001	0.395

**Table 2:** TAS, TOS, OSI, MDA, GSH, and SOD levels in tissue and comparison among rat groups.

Biomarkers	Rat Groups				Pairwise Comparison *p*-Value					
	SG (1)	LPSG (2)	PLPSG (3)	PG (4)	1 vs. 2	1 vs. 3	1 vs. 4	2 vs. 3	2 vs. 4	3 vs. 4
TAS (mmol Eq/g prot)	0.74 ± 0.23	0.13 ± 0.09	0.61 ± 0.19	1.14 ± 0.36	<0.01	0.808	<0.05	<0.05	<0.001	<0.01
TOS (µmol Eq/g prot)	3.42 ± 1.23	9.78 ± 3.88	5.02 ± 0.55	3.34 ± 0.90	<0.001	0.563	1.000	<0.01	<0.001	0.522
OSI	0.53 ± 0.28	12.30 ± 13.43	0.89 ± 0.30	0.33 ± 0.16	<0.05	1.000	1.000	<0.05	<0.05	0.999
MDA (nmol/mg prot)	1.13 ± 0.40	3.38 ± 1.33	1.34 ± 0.61	1.07 ± 0.37	<0.001	0.967	0.999	<0.001	<0.001	0.928
GSH (µmol/g prot)	1.69 ± 0.22	0.39 ± 0.31	1.21 ± 0.37	1.73 ± 0.32	<0.001	0.072	0.994	<0.001	<0.001	<0.05
SOD (U/mg prot)	340.78 ± 69.84	22.35 ± 14.96	207.63 ± 173.46	267.24 ± 74.74	<0.001	0.135	0.597	<0.05	<0.01	0.738

**Table 3:** Comparison of serum CK-MB, CRP, and cTn-I levels among rat groups.

Biomarkers	Rat Groups				Pairwise Comparison *p*-Value					
	SG (1)	LPSG (2)	PLPSG (3)	PG (4)	1 vs. 2	1 vs. 3	1 vs. 4	2 vs. 3	2 vs. 4	3 vs. 4
CK-MB (ng/mL)	9.08 ± 1.08	16.59 ± 3.08	11.95 ± 2.35	8.80 ± 1.86	<0.001	0.147	0.996	<0.01	<0.001	0.098
CRP (ng/mL)	2.99 ± 0.43	6.77 ± 2.19	5.20 ± 2.03	2.89 ± 0.70	<0.01	0.096	0.999	0.328	<0.01	0.077
cTn-I (ng/mL)	57.21 ± 49.92	724.16 ± 277.23	235.63 ± 68.37	58.51 ± 22.07	<0.001	0.179	1.000	<0.001	<0.001	0.184

**Table 4:** Histopathological findings and comparison between groups.

Histopathological Status	Groups				Pairwise Comparison *p*-Value					
	SG (1)	LPSG (2)	PLPSG (3)	PG (4)	1 vs. 2	1 vs. 3	1 vs. 4	2 vs. 3	2 vs. 4	3 vs. 4
Fibrillar tears	0.16 ± 0.40	2.66 ± 0.51	1.66 ± 0.51	0.16 ± 0.40	<0.001	<0.001	1.000	<0.01	<0.001	<0.001
Edema	0.16 ± 0.40	2.66 ± 0.51	1.66 ± 0.51	0.33 ± 0.51	<0.001	<0.001	0.935	<0.05	<0.001	<0.01
Connective tissue enlargement	0.16 ± 0.40	2.50 ± 0.54	1.16 ± 0.40	0.33 ± 0.51	<0.001	<0.01	0.928	<0.001	<0.001	<0.05

## Data Availability

The original contributions presented in this study are included in this article. Further inquiries can be directed at the corresponding author.
